# The effectiveness of topical colloidal silver in recalcitrant chronic rhinosinusitis: a randomized crossover control trial

**DOI:** 10.1186/s40463-017-0241-z

**Published:** 2017-11-25

**Authors:** John R. Scott, Rohin Krishnan, Brian W. Rotenberg, Leigh J Sowerby

**Affiliations:** 1 Department of Otolaryngology, Head and Neck Surgery, Schulich School of Medicine and Dentistry, Western University, London, ON Canada; 2 St. Joseph’s Healthcare, Western University, 268 Grosvenor Street, London, ON N6A 4V2 Canada

**Keywords:** Colloidal silver, Chronic rhinosinusitis, Recalcitrant, Effectiveness, Nasal spray, Topical

## Abstract

**Background:**

Recalcitrant chronic rhinosinusitis without polyposis (CRSsP) is a challenging condition to manage as traditional medical therapies and surgery fail to provide satisfactory clinical improvements. Colloidal silver (CS), a widely used naturopathic agent, has recently shown anti-biofilm properties both in vitro and within a rhinosinusitis animal model. To date, no trials involving humans have been published in world literature. The purpose of this study was to assess the efficacy of CS as a topical nasal spray in patients with refractory CRSsP.

**Methods:**

A prospective cohort study was conducted using a convenience sample of 20 randomized patients with crossover methodology, comparing nasal sprays with CS versus saline. Patients sprayed twice daily for six weeks with the first intervention and then switched to the second for the next six weeks, with measurements made at baseline and each time point. Primary outcomes were changes in SNOT-22 and Lund-Kennedy (LK) endoscopic scores. All analysis was non-parametric and was conducted using STATA 14.

**Results:**

Twenty-two patients were enrolled in the study with 20 completing the entire protocol. Mean 6-week change in SNOT-22 scores were −2.8 and 1.0 for saline and CS, respectively (*p* = 0.373). Similarly, mean 6-week change in LK scores were −1.4 and −1.1 for saline and CS, respectively (*p* = 0.794). Significant period effects were observed with the SNOT-22 score between the randomized groups. No participants experienced negative health effects directly attributable to the administration of intranasal CS.

**Conclusion:**

Commercially available CS nasal spray did not demonstrate any meaningful subjective or objective improvements in patients with recalcitrant CRSsP.

**Trial registration:**

NCT02403479. Registered on March 1, 2015.

## Background

Chronic rhinosinusitis (CRS) is a persistent and debilitating condition affecting up to 15% of the general population [1]. The disease is particularly difficult to manage, with saline lavage, antibiotics and topical intranasal steroids forming the mainstay of medical therapies offered [1, 2]. For the subset of patients who fail conservative treatment strategies surgical intervention is typically recommended [1, 2]. Unfortunately, despite all these efforts, between 6% and 10% of patients will continue to exhibit a recalcitrant form of CRS [3]. Predicting which patients will have treatment resistant CRS is challenging and has not yet been completely characterized in the literature but bacterial biofilms and changes to the microbiome are believed to play an important role [4].

A biofilm can be defined as community of bacteria encased in a protective extracellular matrix, which evades host immune responses and antimicrobial medications [5]. Biofilms have been documented in the sinuses of CRS patients for well over a decade now by many different authors [6–8]. The reported prevalence of biofilms ranges somewhat within CRS populations but was quoted to be as high as 75% in a recent review published by Tatar et al. [9]. Interestingly, *Staphylococcus aureus* (*S. aureus*) has been shown to be not only the dominant biofilm isolate [10], but biofilms consisting of it result in a more relentless disease course amongst CRS sufferers [11].

Silver is one of the most toxic elements to microorganisms and has been a known disinfectant for centuries [12]. During this time, it has seen widespread use across both clinical disease applications and non-medical domains [13, 14]. Silver exhibits activity against Gram-positive and Gram-negative organisms, fungi, protozoa and even some viruses [15]. Coupled with the rise in antibiotic-resistant bacteria, silver has gained headway as a treatment strategy for chronic infections elsewhere in the body. Colloidal silver (CS) spray is a widely used naturopathic product that has sparked research interest because anecdotally individuals with recalcitrant CRS spraying it intranasally experienced an improvement in their symptoms. Commercially available CS, which is simply silver nanoparticles dispersed throughout an aqueous solution, has been noted to attenuate *S. aureus* biofilms in vitro [16]. This report led to an animal model study with sheep that demonstrated safety and continued antibiofilm effects for topical CS at concentrations between 5 and 30 ppm [17]. To date, however, no human trials have been published that examine CS use in refractory CRS.

The purpose of this study was to assess the efficacy of CS as a topical nasal spray in patients with recalcitrant CRS. Secondary aims included monitoring the safety and tolerability of intranasal CS across participants.

## Methods

### Participants

The study was a prospective, double-blinded cohort with crossover methodology conducted at Western University. Blinded patients acted as their own controls by first using an unmarked nasal spray bottle composed of either saline (SL) or colloidal silver (CS) in a randomized fashion, and then switched to the remaining bottle for the second half of the study protocol. The institutional ethics board at Western University granted approval for the investigation. The project was also registered online as a clinical trial (NCT02403479). Patients were approached between January and December 2016 and considered for enrollment if they met diagnostic criteria for chronic rhinosinusitis without polyposis (CRSsP) as defined by the 2011 Canadian clinical practice guidelines and their disease was poorly controlled [1]. Recalcitrant CRSsP was characterized as symptomatic patients with a history of failing functional endoscopic sinus surgery and at least one of the following: failed oral antibiotic therapy; failed topical and/or oral steroid therapy; failed baby shampoo nasal irrigation therapy; failed topical manuka honey therapy. These individuals who were contemplating revision surgery were offered enrollment into the study. Exclusion criteria were individuals with any one of the following: existing autoimmune disorders including diabetes; <18 years of age; an allergy to silver; previous use of CS nasal spray; patients who were pregnant, trying to become pregnant or breastfeeding.

### Randomization

Participants were randomized to receive either 10 ppm CS (Sovereign Silver®; Natural Immunogenics Corp; Sarasota, FL) or saline control intranasal spray for 6 weeks before switching to the other bottle for an additional 6 weeks. Two groups were subsequently organized: Group 1 were those that received SL spray first followed by CS spray after, and Group 2 were patients that used CS spray first and SL spray second. A confidential randomization list was created prior to commencement of the study using a random number generator (Sealed Envelope Ltd.; London, UK). Patients delivered 4 sprays intranasally twice daily from the assigned bottle while entered in the study. All other topical and oral medications that subjects may have been using for their sinus disease were discontinued throughout the duration of the study. A washout period of 2 weeks was implemented between the cessation of maintenance therapies and the study start date. Both the patient and investigators were blinded as to the order of the spray bottles. The CS and SL solutions were indistinguishable from one another in regards to appearance and smell. Participants were reviewed again in clinic at the half way mark (6 weeks) and upon completion of the study protocol (12 weeks). The first spray bottle was exchanged for the second at the 6-week appointment.

### Outcomes

The primary subjective outcome was the 22-item Sino-Nasal Outcome Test (SNOT-22) change from baseline [18]. A Minimal Clinically Important Difference (MCID) change of 9 or more in the SNOT-22 score was considered to be clinically meaningful as this is the smallest difference that can be detected by a patient [18]. Lund-Kennedy endoscopic scores were used as the primary objective outcome, and compared from before and after treatment with a given spray bottle [19]. These parameters were collected at 0, 6 and 12 weeks. Note that the scores at the 6-week mark served as the post-treatment numbers for the first spray and the pre-treatment scores for the second bottle. Patients were also asked to complete a diary to help encourage compliance and to allow them to record any effects, positive or negative, experienced while using the designated nasal sprays. A formal sample size calculation was not performed as no previous studies for intranasal colloidal silver were available. This was a pilot study and 20 participants were enrolled to examine safety, tolerance and possible trends for consideration of a more formal randomized clinical trial.

### Data collection

Descriptive characteristics, including demographics (age, sex), time since diagnosis of CRSsP, chief symptoms, relevant comorbidities (smoking, asthma, environmental allergies), current and past therapies (oral or topical antibiotic and steroid use), presence and number of previous sinus surgeries, and time to follow-up, were collected. After agreeing to participate and a sufficient washout period, patients were seen back in clinic (Week 0) and formally enrolled in the trial. At this visit, participants completed the SNOT-22 questionnaire and nasal endoscopy was performed. A research team member (BWR or LJS), blinded to the spray bottle allocation, determined the Lund-Kennedy endoscopic score.

Following 6 weeks of treatment patients returned to clinic (Week 6) for assessment. The SNOT-22 and Lund-Kennedy scores were recorded in an analogous fashion. Participants were also asked to bring in their diaries to verify compliance and discuss any changes experienced. They were then provided with the second spray bottle that would be used for the next 6 weeks in an identical manner until their last follow-up appointment (Week 12). When patients arrived for their final clinic visit the SNOT-22 and Lund-Kennedy scores were collected and their diary was reviewed.

### Statistical analysis

Statistical analyses were performed using STATA 14 software (StataCorp LLC; College Station, TX). Descriptive statistics were assessed for demographic and clinical data. We captured individual SNOT-22 and Lund-Kennedy scores at baseline, 6 weeks and 12 weeks. Six-week differences in SNOT-22 and Lund-Kennedy scores for both treatments in Group 1 and Group 2 were calculated. For the CS treatment, differences in SNOT 22 and Lund-Kennedy scores were calculated from 6 weeks to 12 weeks in Group 1 and baseline to 6 weeks in Group 2. For the saline treatment, differences in SNOT-22 and Lund-Kennedy scores were calculated from baseline to 6 weeks in Group 1 and 6 weeks to 12 weeks in Group 2. To assess if there was a difference between CS and saline, differences in SNOT-22 and Lund-Kennedy scores were pooled across groups and a *t*-test was used to assess for significance. Unequal variance was assumed. Period effects were determined by testing the differences in the 6 week change in SNOT-22 and Lund-Kennedy scores within Group 1 and Group 2 with a two-sample *t*-test.

## Results

### Study overview

**Fig. 1:**
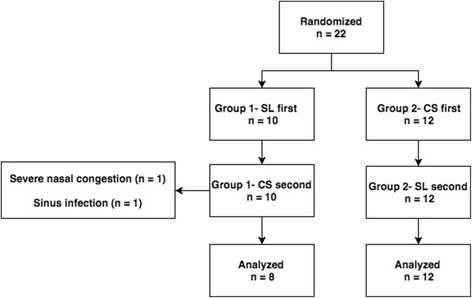
Patient flow diagram. CS- colloidal silver; SL- saline

A total of 22 patients were randomized as depicted in Fig. 1. In Group 1 (*n* = 10), two patients withdrew from the study during the second 6 weeks of the trial, and both withdrew while using the CS nasal spray. The first patient withdrew because of severe nasal obstruction and congestion that was subjectively worse during the CS phase. This effect resolved shortly with discontinuation of the CS therapy and no other interventions were required. The second patient developed an acute exacerbation of sinusitis and removed themselves from the study. The infection improved with oral antibiotics and steroid therapy as prescribed by the patient’s primary care physician. Both of these participants were excluded from final analysis. All patients in Group 2 (*n* = 12) completed the entire study.

### Descriptive statistics

Twenty patients were included in the final analysis. Table 1 outlines the descriptive characteristics for all study participants. The majority of patients had sinonasal symptoms for longer than 5 years and over half had environmental allergies. The mean number of surgeries for all patients enrolled was greater than two and no patient was actively smoking at the time of enrollment. All participants except one were using a form of topical saline spray or irrigation at the time of enrollment. 94.4% and 95.0% of participants had also used oral steroid and oral antibiotic within the previous 12 months respectively. Baseline SNOT-22 and Lund-Kennedy scores were comparable between Groups 1 and 2. A total of 2 patients used oral steroids and 1 patient took oral antibiotics while participating in the study.

**Table 1:** Baseline patient demographics and clinical data

	Group 1 (*n* = 8)	Group 2 (*n* = 12)
Mean age (range in years)	65.8 (44–86)	62.3 (45–84)
Female, n (%)	6 (75.0)	2 (16.7)
CRS symptoms ≤5 years, n (%)	1 (12.5)	2 (16.7)
CRS symptoms 6 or more years, n (%)	7 (87.5)	10 (83.3)
Current smoker, n (%)	0 (0)	0 (0)
Past smoker, n (%)	3 (37.5)	4 (33.3)
Asthma, n (%)	3 (37.5)	4 (33.3)
Environmental allergies, n (%)	6 (75.0)	6 (50.0)
Number of surgeries, mean (SD)	2.5 (1.9)	3.1 (2.5)
Current topical saline irrigation, n (%)	7 (87.5)	12 (100.0)
Oral steroid use last 12 months, n (%)	7 (87.5)	10 (83.3)
Oral steroid use during study, n (%)	1 (12.5)	1 (8.3)
Oral antibiotic use last 12 months, n (%)	8 (100.0)	11 (91.7)
Oral antibiotic use during study, n (%)	1 (12.5)	0 (0.0)
Baseline SNOT-22 score, mean (SD)	59.3 (19.4)	52.8 (19.2)
Baseline LK score, mean (SD)	7.8 (2.2)	6.8 (2.3)

*CRS* chronic rhinosinusitis, *SD* standard deviation, *SNOT-22* Sino Nasal Outcome Test, *LK*- Lund-Kennedy

### Outcome measures

Table 2 depicts the mean SNOT-22 and Lund-Kennedy score at each follow-up time point in the study for Groups 1 and 2. Table 3 summarizes the mean changes in primary outcomes for CS and saline within their respective groups. Notably, in Group 1 the average SNOT-22 score change was −13.9 with the saline bottle but with the CS spray it was +3.9. Upon comparing these values within Group 1 for SNOT-22 score there was no statistical difference (*p* = 0.06), however, when comparing the change in SNOT-22 score *between* the two groups, significant period effects were found (*p* < 0.05). These period effects were not witnessed with the mean Lund-Kennedy score between groups. When the order of spray bottles was ignored and the results were compared across all patients (Table 4), there was no significant difference in SNOT-22 or Lund-Kennedy score between CS and control. Further analysis exclusively comparing outcome measures at 12 weeks to baseline did not demonstrate any clinically or statistically relevant data. In summary, CS did not show any benefit over saline in our study.

**Table 2:** Mean SNOT-22 and LK scores for Groups 1 and 2 at each time point

		Group 1	Group 2
SNOT-22 score, mean (SD)	Baseline	59.3 (19.4)	52.9 (19.2)
	6 weeks	45.4 (16.8)	51.8 (24.4)
	12 weeks	49.3 (17.9)	56.5 (23.5)
LK score, mean (SD)	Baseline	7.8 (2.2)	6.8 (2.3)
	6 weeks	5.9 (1.6)	5.7 (2.8)
	12 weeks	4.8 (2.7)	4.7 (1.6)

*SNOT-22* Sino Nasal Outcome Test, *LK* Lund-Kennedy, *SD* standard deviation

**Table 3:** Mean change in SNOT-22 and LK scores for CS and saline within Groups 1 and 2

		Saline	Colloidal Silver
Change in SNOT-22, mean (SD)	Group 1	−13.9 (16.8)	3.9 (14.7)
	Group 2	4.7 (6.3)	−1.0 (8.1)
Change in LK, mean (SD)	Group 1	−1.9 (2.1)	−1.1 (2.0)
	Group 2	−1.0 (3.6)	−1.1 (3.5)

*CS* colloidal silver, *SNOT-22* Sino Nasal Outcome Test, *LK* Lund-Kennedy

**Table 4:** Pooled 6-week changes in SNOT-22 and LK score results comparing CS versus saline

	Saline	Colloidal silver	*p* value
SNOT-22 score, mean (CI)	−2.8 (−9.6 to 4.1)	1.0 (−4.3 to 6.2)	0.373
LK score, mean (CI)	−1.4 (−2.8 to 0.1)	−1.1 (−2.5 to 0.3)	0.794

*CS* colloidal silver, *SNOT-22* Sino Nasal Outcome Test, *LK* Lund-Kennedy, *CI* 95% confidence interval

### Tolerability

No serious adverse medical events occurred with participants during this investigation. Two patients withdrew from the study, both of who were using the CS nasal spray. One patient experienced profound nasal congestion and another developed a sinus infection but otherwise no major systemic problems were encountered. There were no cases of argyria and, specifically, no bluish discoloration of the sinonasal mucosa was observed. Participant reported compliance rates were high throughout the study with both the saline and CS.

## Discussion

This randomized crossover control trial compared topical CS with saline nasal spray as an adjuvant treatment for patients with recalcitrant CRSsP. It is the first investigation of its kind to examine the effects of CS on the sinonasal cavity of humans. No benefit in SNOT-22 and Lund-Kennedy scoring was recognized for CS, which leads us to question its use as an alternative medicine product for sinus disease. We recognize that the enrolled patient population had particularly challenging rhinosinusitis with the frequent presence of comorbid conditions and use of steroid and antibiotic therapies (Table 1). Twelve of twenty patients had environmental allergies and given known seasonal patterns in CRS exacerbations there was potential for our results to be influenced by seasonal trends [20]. Our trial was run over the course of an entire year, however, so this may have been adjusted for on its own but nevertheless we feel it warrants consideration. The choice to use a 6-week follow-up was made because study participants stopped taking all of their regular sinonasal therapies and if end points were drawn out longer compliance may have become an issue. As it stands, there were no problems with patient compliance in our trial.

Research into the safety profile of CS is limited mainly to animal models and case reports [21]. Indiscriminant use of silver can cause argyria, which is a permanent blue-grey discoloration of the skin, mucosa or internal organs [22]. Although it is not yet known which dose of silver is required to cause argyria, all reports to date are following excessive daily ingestion over a period of years [23, 24]. Argyria is thought to be a benign, cosmetic change in the appearance of tissues and is generally not associated with serious complications. Apart from argyria, extreme cases of systemic silver toxicity can lead to thrombocytopenia, abnormal clotting, renal impairment, proteinuria, and neurological symptoms such as seizures and loss of coordination [22]. Participants in our study did not reveal any signs of argyria but if the CS nasal spray was continued beyond 6 weeks it is certainly possible. We did not monitor blood levels of silver or other serum markers in this investigation given the short duration of exposure and dosing regimen. Rajiv et al. published on a CRS sheep model that saw them irrigate 100 mL of a 30 ppm CS solution daily into the animal’s frontal sinus for 14 days [17]. The animals in their study did not show any health problems during monitoring and they were exposed to dramatically higher cumulative amounts of elemental silver than subjects in our investigation. It is worth again noting that the two patients who withdrew from our study were using the CS nasal spray, and none withdrew from the control group.

This study is inherently limited by its small sample size. Another drawback stems from the delivery method of the medication into the sinuses. We used a low-volume nasal spray rather than a high-volume irrigation, which could have led to unreliable drug delivery. Evidence-based recommendations support high-volume rinses as they maximize therapeutic distribution [25], however silver was not available in this format. An additional limitation of this trial surrounds the use of a commercially available CS product. With this there may be variance in silver concentration between bottles and additional undeclared substances could have been present within them. The concentration of the colloidal silver was not verified by laboratory testing in this investigation. We chose not to exclude certain co-morbid conditions (asthma, ASA sensitivity, etc.) because CRS is a heterogeneous disease and we wanted our study population to resemble a real recalcitrant patient group as much as possible. The bulk of published work supporting the potential of CS in CRS focuses on the presence of *S. aureus* biofilms [4–11]. Although this is the most common biofilm isolate from the paranasal sinuses, we did not formally quantify the presence of this bacterium in our patients. Finally, the results may not be generalizable to all patients with CRS. Our patients had refractory CRS and were collected from a tertiary rhinology center. This means that CS may well provide betterment in a population with less severe CRS.

## Conclusion

This novel study tested CS versus saline nasal spray in patients with recalcitrant CRSsP. We did not demonstrate any statistically significant changes in SNOT-22 or Lund-Kennedy scores over 6 weeks of therapy with CS as compared to control. Period effects were observed between the randomized groups but this did not have any meaningful relevance clinically. Commercially available CS nasal spray does not provide subjective or objective improvements among individuals with treatment resistant CRSsP. Future studies should be larger, employ high-volume irrigations, and examine patients with less severe sinus disease. There still may be a role for CS in CRS populations but we were unable to make a case for it in this study.

## Abbreviations

CI: Confidence interval
CRS: Chronic rhinosinusitis
CRSsP: Chronic rhinosinusitis without polyposis
CS: Colloidal silver
LK: Lund-Kennedy
MCID: Minimal clinically important difference
SD: Standard deviation
SL: Saline
SNOT-22: Sino-nasal outcome test
